# Sublingual immunotherapy: time to reconsider compliance and adherence

**DOI:** 10.1186/1939-4551-8-S1-A68

**Published:** 2015-04-08

**Authors:** Peter Friedrich Eberle

**Affiliations:** 1 Private office, Kassel, Hesse, Zipcode 34121, Wilhelmshöher Allee 109, Germany

## Background

Quality of life (QoL) in patients treated with sublingual-immunotherapy (SLIT) is assessed and compared to QoL in general population aiming to challenge the thesis that non-perception of efficacy by the patient is one of the main causes for poor adherence in SLIT.

## Methods

We conducted a non-interventional, cross-sectional and retrospective quality-of -life- survey including 201 patients treated with SLIT at one pediatric practice in Germany. Clinical or demographical characteristics of the examined patients were not interrogated. Patients´ compliance was calculated based on the duration of active treatment participation. Quality of life was assessed with the generic SF-12 health survey in German language. The items interrogated within the SF-12 health survey are weighted, added up and transformed to a physical component score (PCS) and a mental component score (MCS). The component scores range from 0 to 100, the higher the score the better QoL is perceived.

## Results

Patients´ compliance with SLIT between 2009 and 2014 resulted in a continuation of the treatment by 76-82% in the second and by 60 – 70% in the third year of therapy. The interrogated patients who have been treated with SLIT show a PCS-12 of 49,3 (± 7,0) and a MCS-12 of 52,6 (± 7,2). A different study examining the health status of the German general population (n=2453) results in a PCS-12 of 49,6 (± 8,7) and a MCS-12 of 52,3 (± 8,0) [1] . While patients suffering from perennial or seasonal allergic rhinitis and asthma have significant lower QoL than their control group [2].These observations indicate that patients treated with SLIT have a comparable QoL as the average person in Germany. This outcome is challenging the common opinion of studies reasoning poor adherence of SLIT patients in a non-perception of efficacy.

## Conclusions

This study demonstrates that patients’ compliance with SLIT can be generally high if holistic approaches as strict monitoring are considered. QoL in patients treated with SLIT is as good as QoL of the general population. Poor adherence in SLIT patients cannot be explained by non-perception of efficacy.
